# A Trial of Favipiravir and Hydroxychloroquine combination in Adults Hospitalized with moderate and severe Covid-19: A structured summary of a study protocol for a randomised controlled trial

**DOI:** 10.1186/s13063-020-04825-x

**Published:** 2020-10-31

**Authors:** Mohammad Bosaeed, Ebrahim Mahmoud, Mohammad Hussein, Ahmad Alharbi, Abdulrahman Alsaedy, Adel Alothman, Majed Aljeraisy, Hajar Alqahtani, Marwan Nashabat, Badriah Almutairi, Manar Almaghaslah, Omar Aldibasi, Sameera AlJohani, Abderrezak Bouchama, Yaseen Arabi, Ahmad Alaskar

**Affiliations:** 1 Department of Medicine, King Abdulaziz Medical City, Riyadh, Saudi Arabia; 2 College of Medicine, King Saud bin Abdulaziz University for Health Sciences, Riyadh, Saudi Arabia; 3 King Abdullah International Medical Research Center, Riyadh, Saudi Arabia; 4 Pharmaceutical Care Department, King Abdulaziz Medical City, Riyadh, Saudi Arabia; 5 Intensive Care Department, King Abdulaziz Medical City, Riyadh, Saudi Arabia

**Keywords:** COVID-19, Randomised controlled trial, protocol, Favipiravir, Hydroxychloroquine, Saudi Arabia

## Abstract

**Objectives:**

The selected combination was based on limited evidence clinically and in vitro on the efficacy of the Favipiravir and Hydroxychloroquine in SARS-CoV-2. The two medications were listed in many guidelines as treatment options and ongoing trials assessing their efficacy and safety. Thus, we want to prove the clinical effectiveness of the combination as therapy.

**Trial design:**

This is an Open label, multicenter, randomized controlled clinical trial to evaluate the safety and efficacy of novel therapeutic agents in hospitalized adults diagnosed with COVID-19. It is a multicenter trial that will compare Favipiravir plus Hydroxychloroquine combination (experimental arm) to a control arm.

**Participants:**

All study procedures will be conducted in eight centres in Saudia Arabia:

King Abdulaziz Medical City National Guard Health Affairs in Riyadh.

King Abdulaziz Hospital - Al Ahsa, Saudi Arabia

AlMadina General Hospital, Madnia, Saudi Arabia

Al-Qatif Central Hospital, Saudi Arabia

Imam Abdulrahman Al Faisal Hospital, Dammam, Saudi Arabia

King Abdulaziz Medical City, Jeddah, Saudi Arabia

King Abdulaziz Hospital, Makkah, Saudi Arabia

Imam Abdulrahman Alfaisal Hospital, Riyadh, Saudi Arabia

Inclusion Criteria

• Should be at least 18 years of age,

• Male or nonpregnant female,

• Diagnosed with COVID-19 by PCR confirmed SARS-coV-2 viral infection.

• Able to sign the consent form and agree to clinical samples collection (or their legal surrogates if subjects are or become unable to make informed decisions)..

• Moderate or Severe COVID-19, defined as oxygen saturation (Sao2) of 94% or less while they were breathing ambient air or significant clinical symptoms that require hospital admission.

• patients had to be enrolled within 10 days of disease onset.

Exclusion Criteria

• Patients who are pregnant or breastfeeding.

• Will be transferred to a non-study site hospital or discharged from hospital within 72 hours.

• Known sensitivity/allergy to hydroxychloroquine or Favipiravir

• Current use of hydroxychloroquine for another indication

• Prior diagnosis of retinopathy

• Prior diagnosis of glucose-6-phosphate dehydrogenase (G6PD) deficiency

• Major comorbidities increasing the risk of study drug including: i. Hematologic malignancy, ii. Advanced (stage 4-5) chronic kidney disease or dialysis therapy, iii. Known history of ventricular arrhythmias, iv. Current use of drugs that prolong the QT interval, Severe liver damage (Child-Pugh score ≥ C, AST> 5 times the upper limit), HIV.

• The investigator believes that participating in the trial is not in the best interests of the patient, or the investigator considers unsuitable for enrollment (such as unpredictable risks or subject compliance issues).

• Clinical prognostic non-survival, palliative care, or in deep coma and no have response to supportive treatment within three hours of admission

• Patient with irregular rhythm

• Patient with a history of heart attack (myocardial infarction)

• Patient with a family history of sudden death from heart attack before the age of 50

• Take other drugs that can cause prolonged QT interval

• Patient who is receiving immunosuppressive therapy (cyclosporin) which cannot be switched to another agent or adjusted while using the investigational drug

• Gout/history of Gout or hyperuricemia (above the ULN), hereditary xanthinuria or xanthine calculi of the urinary tract.

**Intervention and comparator:**

The treatment intervention would be for a maximum of 10 days from randomization and it would be as follows:

Favipiravir for 10 days: Administer 1800 mg (9 tablets) by mouth twice daily for one day, followed by 800mg (4 tablets) twice daily (total days of therapy is 10 days)

Hydroxychloroquine for 5 days: (400mg) twice daily on day 1; for days 2-5 (200mg) twice daily.

Reference Comparator Therapy:

Standard of care is defined as: Treatment that is accepted by medical experts as a proper treatment for Covid-19 disease. Standard care comprised of, as necessary, supplemental oxygen, noninvasive and invasive ventilation, antibiotic agents, vasopressor support, renal-replacement therapy, extracorporeal membrane oxygenation (ECMO), and antiviral therapy except Favipiravir. Also, it may include intravenous fluids and medications for symptoms relief .

**Main outcomes:**

The primary endpoint is the time to clinical improvement, defined as the time from randomization to an improvement of two points (from the status at randomization) on a seven-category ordinal scale or live discharge from the hospital, whichever came first (14 days from Randomization).

**Randomisation:**

Eligible participants will be randomized in a 1:1 ratio to either the combination group (Favipiravir and Hydroxychloroquine) or a control group. The patients will be randomized utilizing Web based data entry System with a stratification based on the centre and the ICU admission.

**Blinding (masking):**

This is an Open label study and only the analyst will be blinded during the study conduct.

**Numbers to be randomised (sample size):**

Under the classical two arm parallel design the total effective sample sizes needed is 472 subjects (236 subjects per group).

**Trial status:**

Protocol version 3.1 (dated 11 Aug 2020), and currently recruitment is ongoing.

The date recruitment started was May 21, 2020 and the investigators anticipate the trial will finish recruiting by the end of December 2020.

**Trial registration:**

ClinicalTrials.gov Identifier: NCT04392973, 19 May 2020

**Full protocol:**

The full protocol is attached as an additional file, accessible from the Trials website (Additional file 1). In the interest in expediting dissemination of this material, the familiar formatting has been eliminated; this Letter serves as a summary of the key elements of the full protocol.

**Supplementary information:**

**Supplementary information** accompanies this paper at 10.1186/s13063-020-04825-x.

## Supplementary information

**Additional file 1.** Full Study Protocol.

## Data Availability

The corresponding author has access to the final trial information, and the data will be available on reasonable request (Contact: dr.bosaeed@live.com).

