# Supplementary material for: A Trial of Favipiravir and Hydroxychloroquine combination in Adults Hospitalized with moderate and severe Covid-19: A structured summary of a study protocol for a randomised controlled trial
Source: Trials. 2020 Oct 31;21:904. doi: 10.1186/s13063-020-04825-x (PMC7602769; doi:10.1186/s13063-020-04825-x)
Supplement: Supplementary file 1 — Additional file 1. Full Study Protocol. [file 13063_2020_4825_MOESM1_ESM.pdf]

RYD-20-419812-52930

King Abdullah International Medical Research Center
KAIMRC
APPROVED
IRB
Institutional Review Board

A Trial of Favipiravir and Hydroxychloroquine combination in Adults Hospitalized with moderate and severe Covid-19

CLINICAL TRIAL PROTOCOL

**Short Title**	FAvipiravir and HydroxyChloroquine Combination Therapy (FACCT Trial)
**Investigational Drug**	Favipiravir and Hydroxychloroquine
**Protocol No**	RC20/174/R
**Sponsor**	King Abdullah International Medical Research Center

Protocol Version: 3.1

Date: 11 Aug 2020

Confidentiality Statement

This document contains confidential information that must not be disclosed to anyone other than the Sponsor, the Investigator Team, host organization, members of the Research Ethics Committee and other regulatory bodies. This information cannot be used for any purpose other than the evaluation or conduct of the clinical investigation without the prior written consent of Dr. Mohammad Bosaeed.

Statement of Compliance

The trial will be conducted in compliance with the protocol, the principles of Good Clinical Practice (GCP) Guideline, and all other applicable regulatory requirements.

Chief Investigator Approval and Agreement

I have read the trial protocol and agree to conduct the trial in compliance with the protocol, the principles of Good Clinical Practice and all applicable regulatory requirements.

I hereby approve this version of the protocol and declare no conflict of interest

Mohammad Bosaeed

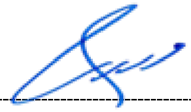

11/08/2020

Chief Investigator

Name Signature Date

STUDY PROTOCOL AGREEMENT FORM

I, Dr. __________________, Site principal investigator, have examined this protocol entitled:

A Trial of Favipiravir and Hydroxychloroquine combination in Adults Hospitalized with moderate and severe Covid-19

I agree to conduct this study according to the protocol and its amendments and to comply with the ICH GCP, Applicable regulations and the requirements of the subject ethical, legal and safety considerations.

Site Investigator Name:

Hospital Name:

Hospital Address:

Date:

Signature:

Principle Investigator Name:

Hospital Name:

SIGNATURE:

TABLE OF CONTENTS

STUDY PROTOCOL AGREEMENT FORM ... 3

Table of contents ... 4

List of Tables ... 6

List of Figures ... 6

Abbreviations ... 7

Protocol Amendment Summary of Changes ... 8

Protocol Summary ... 9

1. Introduction ... 14

1.1. Background ... 14

1.2. Study Medication: ... 15

1.2.1. Favipiravir ... 15

1.2.2. Hydroxychloroquine ... 16

1.3. Dose Adjustment [13,14] ... 16

1.4. Drug interaction ... 17

1.5. Rationale of the study ... 18

2. Study objectives and endpoints ... 24

2.1 Primary Objectives ... 24

2.2 Secondary Objectives ... 24

2.3 Primary Endpoint ... 24

2.4 Secondary Endpoint ... 25

3. STUDY DESIGN ... 25

4. Study Population ... 25

4.1 ... 25

4.1Inclusion Criteria ... 25

4.2 Exclusion Criteria ... 26

4.3 Patient recruitment ... 27

5. Premature discontinuation of the study ... 27

6. Treatments ... 28

6.1 Description of investigational drug and reference therapy ... 28

6.2 Packaging and labelling[13,14] ... 28

6.3 Treatment assignment ... 29

6.4 Randomization ... 29

6.5 Storage conditions 29
6.6 Treatment compliance 30
6.7 Accountability and Destruction 30
6.8 Concomitant treatments 30
7. Procedures and assessments 31
7.1 Screening/Baseline 31
7.2 Treatment Period 31
7.3 Follow-up Period 32
7.4 Laboratory Method 29
8. Flow Chart 34
9 STATISTICAL CONSIDERATIONS 35
9.1 General Considerations 35
9.2 Sample Size and Power Considerations 35
9.3 Statistical Analysis 39
10. Adverse events 40
10.1 Definitions 40
10.2 Adverse event with interventional medication 41
10.3 Handling of Non-Serious Adverse Events 42
10.4 Handling of Serious Adverse Events 43
10.5 Regulatory Responsibility 44
11. Ethical and regulatory standards 44
11.1 Ethics and Good Clinical Practices 44
11.2 Ethics Committee 45
11.3 Informed consent 45
12. Study monitoring, datamanagement and quality assurance 47
12.1 Case Report Forms (CRFs) & Recording of Data 47
12.2 Data Safety Monitoring Board 48
13. Administrative rules 48
13.1 Secrecy Agreement 48
13.2 Protocol amendments 48
13.3 Record retention in investigation centre(s) 49
13.4 Insurance compensation 49
13.5 Sponsor audits and inspections by regulatory agencies 49
13.6 Discontinuation of the study 49
14. Timelines 49
15. Publication 50
Appendix 50
References 50

LIST OF TABLES:

Table 1 Dose Adjustment for study medication 16
Table 2 Packaging and labelling of the study medications... 28
Table 3 Storage Conditions for study medications 29
Table 4: Flow Chart 34

LIST OF FIGURES

Figure 1: Study Design 20
Figure 2 . A: Impact of proportion of events in treatment and control on Power. B: Impact of HR and proportion of events in treatment arm on power... 38

ABBREVIATIONS:

AE	Adverse Event/Adverse Experience
APACHE	Acute Physiology and Chronic Health Evaluation (score)
CRF	Case Report Form
COVID-19	Coronavirus disease 2019
CXR	Chest X-ray
DSMB	Data and Safety Monitoring Board
ECMO	Extra corporeal membrane oxygenation
FDA	Food and Drug Administration
GCP	Good Clinical Practice
HIV	Human immunodeficiency virus
ICH	International Conference on Harmonization
ICU	Intensive Care Unit
IRB	Institutional review board
LFT	Liver function test
MAP	Mean arterial pressure
NP	Nasopharyngeal
PCR	Polymerase chain reaction
qPCR	quantitative polymerase chain reaction
PI	Principle Investigator
PK	Pharmacokinetic
SAE	Serious adverse event
SARS-CoV-2	severe acute respiratory syndrome coronavirus 2
SFDA	Saudi Food and Drug Administration

PROTOCOL AMENDMENT SUMMARY OF CHANGES

Version	Date	Author(s)	Co-Author(s)	Modifications
1.0	7/4/2020	Ebrahim Mahmoud, Majed Aljeraisy, Hajer Alqahtani, Marwan Nashabat, and Mohammad Bosaeed		
2.0	26/4/2020	Mohammad Bosaeed, Ebrahim Mahmoud, Hajar Alqahtani		
3	31/5/2020	Mohammad Bosaeed, Ebrahim Mahmoud, Hajar Alqahtani, and Badriah Amutairi		
3.1	05/07/2020	Mohamed Hussein, Mohammad Bosaeed		Study design and analysis plan, minor change in consenting as per FDA request

PROTOCOL SUMMARY:

Title	A Trial of Favipiravir and Hydroxychloroquine combination in Adults Hospitalized with moderate and severe Covid-19
Short title	FAvipiravir and HydroxyChloroquine Combination Therapy (FACCT Trial)
Protocol Number	RC20/174
Methodology	Multicenter, randomized, parallel groups
Study Center(s)	The study will be conducted in KAMC-Riyadh (other centers will be added upon approval)
Aim of the study	The purpose of this study is to evaluate the clinical efficacy and safety of Favipiravir and Hydroxychloroquine combination in the treatment of moderate and severe cases of COVID-19 disease.

<table>
<tr><td>Objectives</td><td>

<h3>Primary Objectives</h3>

The improvement of two points (from the status at randomization) on a seven-category ordinal scale* or live discharge from the hospital, whichever came first.

*The seven-category ordinal scale consists of the following categories:

1. Not hospitalized with resumption of normal activities
2. Not hospitalized, but unable to resume normal activities
3. Hospitalization, not requiring supplemental oxygen
4. Hospitalization, requiring supplemental oxygen
5. Hospitalization, requiring nasal high-flow oxygen therapy and/or non-invasive mechanical ventilation
6. Hospitalization, requiring ECMO and/or invasive mechanical ventilation
7. Death.

<h3>Secondary Objectives</h3>

1. To evaluate the progress in clinical status of COVID-19 patients in both arms
2. To monitor the viral shedding duration and PCR test conversion days from positive to negative.
3. Evaluate the safety of investigational therapeutics as compared to the control arm.
4. To evaluate difference in the complications and prognosis of COVID-19 patients between the two groups.

</td></tr>
</table>

Inclusion / Exclusion criteria	Inclusion Criteria:<br>1. Should be at least 18 years of age,<br>2. Male or nonpregnant female,<br>3. Diagnosed with COVID-19 by PCR confirmed SARS-coV-2 viral infection.<br>4. Able to sign the consent form and agree to clinical samples collection (or their legal surrogates if subjects are or become unable to make informed decisions).<br>5. Moderate or Severe COVID-19, defined as oxygen saturation (Sao2) of 94% or less while they were breathing ambient air or significant clinical symptoms with Chest X ray changes that require hospital admission.<br>6. Patients had to be enrolled within 10 days of disease onset.<br>7. Not part of any other clinical trial and agrees not to participate in any.<br><br>Exclusion Criteria:<br>1. Patients who are pregnant or breastfeeding.<br>2. Will be transferred to a non-study site hospital or expected to be discharged within 72 hours.<br>3. Known sensitivity/allergy to hydroxychloroquine or Favipiravir<br>4. Current use of hydroxychloroquine for another indication<br>5. Prior diagnosis of retinopathy<br>6. Prior diagnosis of glucose-6-phosphate dehydrogenase (G6PD) deficiency<br>7. Major comorbidities increasing the risk of study drug including: i. Hematologic malignancy, ii. Advanced (stage 4-5) chronic kidney disease or dialysis therapy, iii. Known history of ventricular arrhythmias, iv. Current use of drugs that prolong the QT interval, Severe liver damage (Child-Pugh score ≥ C, AST> 5 times the upper limit), HIV.<br>8. The investigator believes that participating in the trial is not in the best interests of the patient, or the investigator considers unsuitable for enrollment (such as unpredictable risks or subject compliance issues).<br>9. Clinical prognostic non-survival, palliative care, or in deep coma and no have response to supportive treatment within three hours of admission.

	10. Patient with irregular rhythm or the QTc (corrected QT interval) in the baseline ECG more than 490ms.<br>11. Patient with a history of heart attack (myocardial infarction)<br>12. Patient with a family history of sudden death from heart attack before the age of 50<br>13. Take other drugs that can cause prolonged QT interval<br>14. Patient who is receiving immunosuppressive therapy (cyclosporin) which cannot be switched to another agent or adjusted while using the investigational drug<br>15. Gout/history of Gout or hereditary xanthinuria or xanthine calculi of the urinary tract.<br>16. Serum uric acid level > 2 times upper level of normal associated with symptoms of gout
Number of subjects	520 (260 per group).
Study product	Route of Administration: Oral (or through Nasogastric tube)<br><br>Dose:<br>Favipiravir: Administer 1800 mg (9 tablets) by mouth twice daily for one day, followed by 800mg (4 tablets) twice daily (total days of therapy is 10 days or till hospital discharge)<br><br>Hydroxychloroquine (400mg) twice daily on day 1; for days 2-5 (200mg) twice daily.
Duration of administration	Favipiravir: 10 days<br>Hydroxychloroquine: 5 days
Reference therapy	Standard of care

Evaluation criteria	Primary Efficacy Outcome measures:<br><br>The primary endpoint is the time to clinical improvement, defined as the time from randomization to an improvement of two points (from the status at randomization) on a seven-categoryordinal scale or live discharge from the hospital, whichever came first.<br><br>**Secondary Endpoint**<br><br>1 Clinical status assessed by the seven-category ordinal scale.<br>2 The requirement of ICU admission or Mechanical ventilation<br>3 28- and 90-days mortality.<br>4 PCR test negative conversion rate and days from positive to negative**<br>5 Evaluate the safety of investigational therapeutics as compared to the control arm.<br>6 Length of hospital stay.<br>7 Duration of fever.<br>8 QT prolongation (Daily follow up during the concomitant use of both medications and regularly till Day 14)<br><br>Safety Outcome Measures:<br><br>Safety assessments will consist of monitoring and recording all adverse events and serious adverse events occurring during the regular monitoring of hematology and blood chemistry, ECG, regular measurement of vital signs, performance of physical and neurological examinations if needed.
Primary End Point	The time to clinical improvement, defined as the time from randomization to an improvement of two points (from the status at randomization) on a seven-category ordinal scale or live discharge from the hospital, whichever came first
Safety End Points	This study will be able to determine the safety of investigational therapeutics to determine if the rate of AEs is higher, lower, or no different than control arm.

Statistical methodology	Sample size. 520 (260 per group).<br><br>Analysis of Primary Endpoint:<br>The primary endpoint of the current study is Median time to clinical improvement by at least 2 points or discharge from the hospital. The number and percent of subjects who met the endpoint by day 28 of follow up will be calculated and tabulated. Kaplan-Meier plot of time from randomization to endpoint will be generated (censoring at 28 days if completed follow-up, or at date of last contact if lost to follow-up prior to 28 days), with associated table showing: number censored and number (%) meeting the endpoint, median time to endpoint and associated HR with 95% confidence interval; p-value from cox proportional hazard regression.
Study schedule	attached
Study duration<br>/ Timelines	The estimated timelines are the following:<br>IRB Approval .... April 2020<br>Saudi Food & Drugs Authority Approval ....April 2020<br>First Site Initiation Visit ....May 2020<br>First Patient First Visit .... May 2020<br>Last Patient First Visit .... May 2021<br>Last Patient Last Visit ....Nov 2021<br>Database lock ....Nov 2021<br>Final Study Report .... Dec 2021

1. INTRODUCTION

1.1. Background

Beginning in December 2019, a novel coronavirus, designated SARS-CoV-2, has caused an international outbreak of respiratory illness termed Covid-19. The WHO declared the epidemic of COVID-19 as a pandemic on March 12th, 2020 [1]. According to a recent Chinese study, about 80% of patients present with mild disease and the overall case-fatality rate is about 2.3% but reaches 8.0% in patients aged 70 to 79 years[2]. In Saudi Arabia, as of March 27, 2020, 1012 confirmed cases of the disease were reported[2]. Till now, there are no

specific therapeutic agents based on strong evidence, for this novel coronavirus infections, however, several medications have been evaluated as a potential therapy. Therapy warranted not only to treat symptomatic patients but also to decrease the duration of virus carriage in order to limit the transmission in the community.

Favipiravir which is a new type of RNA-dependent RNA polymerase (RdRp) inhibitor which has activity against influenza virus. In addition to its anti-influenza virus activity, favipiravir is capable of blocking the replication of flavi-, alpha-, filo-, bunya-, arena-, noro-, and other RNA viruses[3]. Favipiravir is converted into an active phosphoribosylated form (favipiravir-RTP) in cells and is recognized as a substrate by viral RNA polymerase, thus inhibiting RNA polymerase activity[4] , which theoretically can be active against SARS-CoV-2. Favipiravir compared to lopinavir/ritonavir in a study of 80 patients who has Covid-19, a shorter viral clearance time was found for the Favipiravir arm versus the lopinavir/ritonavir arm (median (interquartile range, IQR), 4 (2.5–9) d versus 11 (8–13) d, $P < 0.001$). The Favipiravir arm also showed significant improvement in chest imaging compared with the lopinavir/ritonavir arm, with an improvement rate of 91.43% versus 62.22% ($P = 0.004$)[5]. Furthermore, it was superior to Arbidol in having a higher 7-day clinical recovery rate in patients with Covid-19 and a more effective in reducing the incidence of fever and cough [1]. While Chloroquine is a widely used antimalarial that was found to be a potential broad-spectrum antiviral in 2006 [6]. Chloroquine was found to block SARS-CoV-2 infection at low-micromolar concentration[7]. Hydroxychloroquine, an analogue of chloroquine, has a clinical safety profile that is better than that of chloroquine and allows higher daily dose[8]. In multicenter clinical trials conducted in China evaluating the efficacy of chloroquine or hydroxychloroquine, results from more than 100 patients have demonstrated that chloroquine phosphate is superior to the control treatment in inhibiting the exacerbation of pneumonia, promoting a virus-negative conversion, and shortening the disease course according to the news briefing[9]. Thus, Hydroxychloroquine was included in many treatment pathways including Saudi Center of Disease and Prevention.

1.2. Study Medication:

1.2.1. Favipiravir

Favipiravir is a selective and potent inhibitor of influenza viral RNA polymerase. It acts as a purine analogue, which selectively inhibits viral RNA-dependent RNA polymerase (RdRps). Based on data from in vitro assay of influenza virus, favipiravir is incorporated into cells, undergoes phosphoribosylation and further phosphorylation to become favipiravir ribofuranosyl phosphates (RTP) which blocks viral RdRp. Hypothesis suggests that

favipiravir-RTP works by blocking polymerase domains, thus preventing incorporation of nucleotides for viral RNA replication and transcription.[4]

Favipiravir has the characteristic of acting on RNA viruses including Ebola and Coronaviruses especially novel coronavirus (2019-nCoV). For Ebola virus, favipiravir was effective in preventing Ebola in mice by 100% although EC50 (drug concentration found to reduce viral replication by 50%) ~67 µM. Recent in vitro study on clinical isolates of 2019nCoV showed that Favipiravir has EC50 =61.88µM. [7]

1.2.2. Hydroxychloroquine

Hydroxychloroquine (HCQ) is an antimalarial agent with anti-inflammatory and immunomodulatory activities which has been gaining great interest recently for it's antiviral activity. Data on using HCQ for Coronavirus is very limited. In vitro analyses on Vero cells demonstrated the potency of hydroxychloroquine in which EC50= 0.72 µM against SARS-CoV-2. [10,11]

HCQ antiviral activity is believed to be related to it's ability to inhibit SARS-CoV entry through changing the glycosylation of ACE2 (Angiotensin Converting Enzyme-2) receptor and spike protein and also inhibits post-entry stages of SARS-CoV-2 by blocking the transport of SARS-CoV-2 from early endosomes to endolysosomes, which appears to be a requirement to release the viral genome. [12]

1.3. Dose Adjustment [13,14]

Table 1 Dose Adjustment for study medication

	**Favipiravir**	**Hydroxychloroquine**
**Renal impairment**	No recommendations for dosage adjustment provided by the manufacturer in package insert	No recommendations for dosage adjustment provided by the manufacturer in package insert

**Hepatic impairment**	Higher plasma levels reported with severe hepatic impairment (Child Pugh Class C).<br>Dosage regimen used for patients with Child Pugh Class C: 800 mg BID for one day, followed by 400 mg BID per package insert	No recommendations for dosage adjustment provided by manufacturer in package insert. Instead, cautionary use was suggested

1.4. Drug interaction

Favipiravir Drug Interactions [14]

- Favipiravir inhibits aldehyde oxidase irreversibly, inhibits CYP2C8 and to a lesser extent CYP1A2, 2C9, 2C19, 2D6, 2E1 and 3A4.
- Drugs that interact with favipiravir:
  - Theophylline increases favipiravir Cmax and AUC by about 30%.
  - Pyrazinamide increases uric acid serum level when administered concomitantly with favipiravir due to increase reabsorption of uric acid in the renal tubules
  - Repaglinide plasma level may increase due to CYP2C8 inhibition
  - Famciclovir plasma level may be decreased due to aldehyde oxidase inhibition
  - Use Favipiravir must be with caution while using acetaminophen – dosing of acetaminophen should be no more than 3000mg/day (or less in patients with hepatic insufficiency)

HYDROXYCHLOROQUINE DRUG INTERACTIONS[13-15]

- HCQ is metabolized by CYP enzymes (CY2D6, 2C8, 3A4, and 3A5) into 3 metabolites:desethylhydroxychloroquine,desethylchloroquine,and bidesethylchloroquine.
- Data from previous study done by the Plaquenil Lupus Systemic Study Group in which authors analyzed blood HCQ concentrations in patients receiving enzyme inducers or inhibitors. They concluded that no pharmacokinetic interaction has been observed between these drugs and blood HCQ concentration based on similar mean plasma drug concentration of HCQ achieved.

- No data are available to evaluate effect of favipiravir (CYP 2C8 inhibitor) on HCQ plasma concentration.
- We concluded that no clear risk of HCQ accumulation when used concomitantly with favipiravir based on data extrapolated from previously mentioned study
- However, we recommend to exercise caution when starting HCQ with following medications.
- Digoxin serum levels may increase with concomitant use of HCQ. Serum digoxin levels should be closely monitored
- Insulin hypoglycemia effect may be enhanced with HCQ. Dosage adjustment of insulin/antidiabetic drugs may be required. Patients treated with hydroxychloroquine should be monitored for hypoglycemia. We recommend frequent blood glucose monitoring. In cases of severe hypoglycemia, hydroxychloroquine should be discontinued.
- Drugs that prolong QT interval and other arrhythmogenic drugs can potentiate QTc prolongation associated with HCQ. HCQ should not be administered with other drugs that have the potential to prolong QTc.
- Antiepileptics drugs effect might be compromised with HCQ due to lower seizure threshold
- Cyclosporin serum level may increase when administered with HCQ. Cyclosporin serum level monitoring is recommended.

1.5. Rationale of the study

There is an urgent need to explore therapeutic options for SARS-CoV-2 in order to face the pandemic. In the current guidelines, HCQ has been recommended for treatment of COVID-19 (MOH and Saudi CDC guidelines) due to the early data supporting the use of this drug off-label, especially during the current emergency circumstance and the lack of clear evidence and licensed therapy. The proposed mechanistic antiviral effect of HCQ in COVID-19 is related to it is ability to inhibit SARS-CoV2 entry through changing the glycosylation of ACE2 (Angiotensin Converting Enzyme-2) receptor and spike protein and also inhibits post-entry stages of SARS-CoV-2 by blocking the transport of SARS-CoV-2 from early endosomes to endolysosomes, which appears to be a requirement to release the viral genome

On the other hand, favipiravir is a potent inhibitor of viral RNA polymerase. Hypothesis suggests that favipiravir-RTP (favipiravir metabolite) works by blocking polymerase domains, thus preventing incorporation of nucleotides for viral RNA replication and transcription.

Theoretically, combining both agents would result into synergistic effect due to different target sites. However, no previous studies have explored this theory yet. In vitro data demonstrate antiviral effect of both agents against COVID-19. Well conducted/established clinical trials are yet to be developed to evaluate the safety and efficacy of both agents among other agents that believed to have antiviral effect against COVID-19. We believed that both agents have reasonable safety profile if used appropriately. Therefore, combining two potential therapeutic options seems to be an attractive approach, taking into consideration that we are targeting patients with moderate to severe COVID-19 were mortality rate reaches up to 20% in severe cases.

Ongoing clinical trials applying a combination of various potential therapies for COVID-19 is a common approach in these situations, such as lopinavir/ritonavir NCT04303299; tocilizumab NCT04310228; chloroquine NCT04319900; and HCQ NCT04303299, among others, which all have not been completed yet.

The selected combination was based on limited evidence clinically and in vitro on the efficacy of the Favipiravir and Hydroxychloroquine in SARS-CoV-2. The two medications were listed in many guidelines as treatment options and ongoing trials assessing their efficacy and safety. Thus, we want to prove the effectiveness of the combination as therapy.

1.5.1. Research hypothesis

We assume superiority of combination therapy compared to standard of care.

Time to discharge or clinical improvement by 2 points in the standard of care arm is expected to be (median: 11 days)[5]. The exact treatment effect from Favipiravir tables + Hydroxychloroquine is not exactly known but can be approximated using prior clinical studies. A study comparing the effect of Favipiravir to lopinavir/ritonavir on virus clearance has shown a 64% reduction in time to viral clearance in the Favipiravir arm. This was equivalent to a median of 11 days. We assume that clinical improvement might take place a little later to viral clearance and therefore we assume that the treatment arm might result in a minimum clinically meaningful expected reduction of 30% (form a median of 11 days to a median of 7.7 days). This is equivalent to a Hazard ratio of 0.7.

We further assume, that 50% of the patients in the control group will have clinical improvement/ discharged and 70% will have clinical improvement/discharged in the treatment arm.

1.5.2. Study Design

This study is a randomized, controlled, open-label trial to evaluate the safety and efficacy of novel therapeutic agents in hospitalized adults diagnosed with COVID-19. It is a multicenter trial that will compare Favipiravir plus Hydroxychloroquine combination (experimental arm) to a control arm.

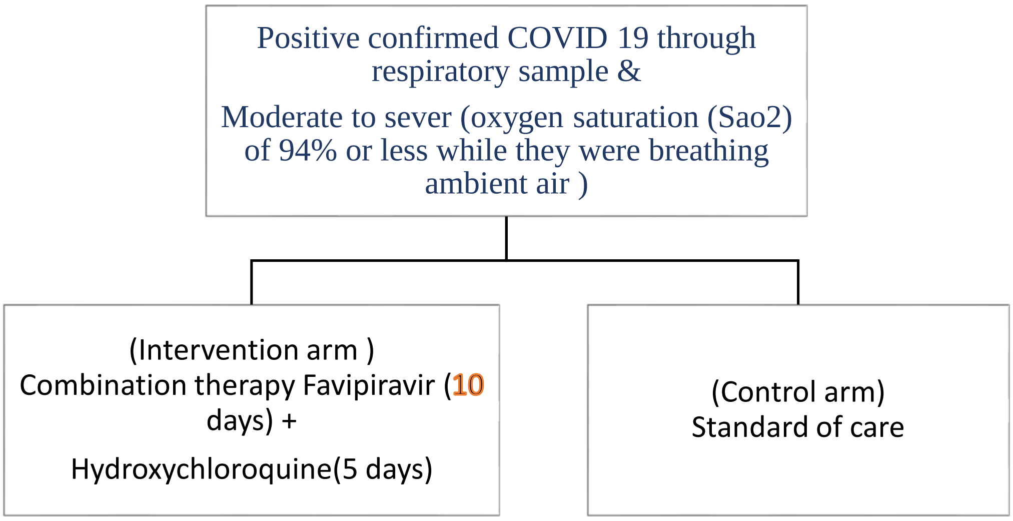

Figure 1: Study Design

1.5.3. Dose selection

FAVIPIRAVIR DOSING AND ADMINISTRATION

Approved dosage for influenza: 1600 mg BID on day1, followed by 600 mg BID on day 2-5.

Suggested dose for COVID-19 by the manufacturer FFTC and the Japanese guideline: 1800 mg BID on day 1 followed by 800 mg BID on day 2-10

Evidence demonstrating safety and efficacy of higher doses for influenza infection:

1. A placebo-controlled phase I/II study in type A or B influenza patients was conducted (1800 mg BID followed by 800 mg BID for 4 days)

a. 1800/800 mg BID regimen resulted in Cmax= 64.56 mcg/mL, AUC= 446 mcg.hr/mL and associated with significant difference in the time to alleviation of influenza symptoms compared to placebo

2. Two placebo-controlled **phase III** studies on type A or type B influenza patients (1800 mg BID on day1 followed by 800 mg BID on day 2-5)

   a. The median time required to alleviate influenza symptoms was 84 hr vs 99hr; P=0.004 for favipiravir and placebo group respectively in study 1

   b. The median time required to alleviate influenza symptoms was 78 hr vs 84 hr; P=0.303 for favipiravir and placebo respectively in study 2

Evidence demonstrating safety and efficacy of higher doses for <u>EBOV infection</u>:

1. Higher concentrations of favipiravir were required to inhibit EBOV than H1N1 based on preclinical studies (150 mg/kg/day vs 100 mg/kg/day in infected mice)
2. In JIKI Trial (prove of concept trial), participants infected with EBOV received oral favipiravir (day0:6,000 mg; day1 to day9:2,400mg/d)
3. Although RNA viral load values and mortality were not significantly different between adults starting favipiravir within <72h of symptoms compared to others. Favipiravir was well tolerated.

<u>Oral Tablet:</u>

Administer 1800 mg (9 tablets) by mouth twice daily for one day, followed by 800mg (4 tablets) twice daily (total days of therapy is 10days)

For patients with Nasogastric tube:

- Prepare the drug suspension by adding 5 ml of warm water to each tablet (simple suspension method; concentration 40 mg/mL).
- Slowly inject the suspension into NG tube using a piston.
- Wash nasogastric tube with 5 mL of water.
- Follow all precaution during preparing suspension due to teratogenic effect observed in animal studies.

HYDROXYCHLOROQUINE DOSING AND ADMINISTRATION

No dosage regimen has been approved for HCQ as antiviral agent, however, a robust data derived from pharmacokinetic study confirmed that the dosing regimen below has demonstrated maximum viral killing activity with lowest toxicity treatment of SARS-CoV-2. [12]

Oral Tablet:

Administer 400 mg (2 tablets) by mouth twice daily for one day, followed by 200mg (1 tablet) twice daily on day 2-5 (total days of therapy is 5 days)

For patients with Nasogastric tube, prepare suspension at bedside as follows:

- Prepare the drug suspension by adding 5 mLs of water to each tablet (simple suspension method; concentration 40 mg/mL).
- Slowly inject the suspension into NG tube using a piston.
- Wash nasogastric tube with 5 mL of water.

There are limited data on hydroxychloroquine teratogenicity. We advice to follow all precaution during preparing suspension

Duration: In one study, **10 days** course of favipiravir was compared to Arbidol and found to be superior in having a higher 7-day clinical recovery rate in patients with COVID-19 and more effective in reducing the incidence of fever and cough. In JIKI Trial, 10 days course of favipiravir was administered

Based on the proposed/hypothetical pathogenesis of COVID-19, the clinical phase is divided into three phases: the viremia phase, the acute phase (pneumonia phase) and the recovery phase. Based on the proposed duration of viremia phase, 10 days treatment is recommended.

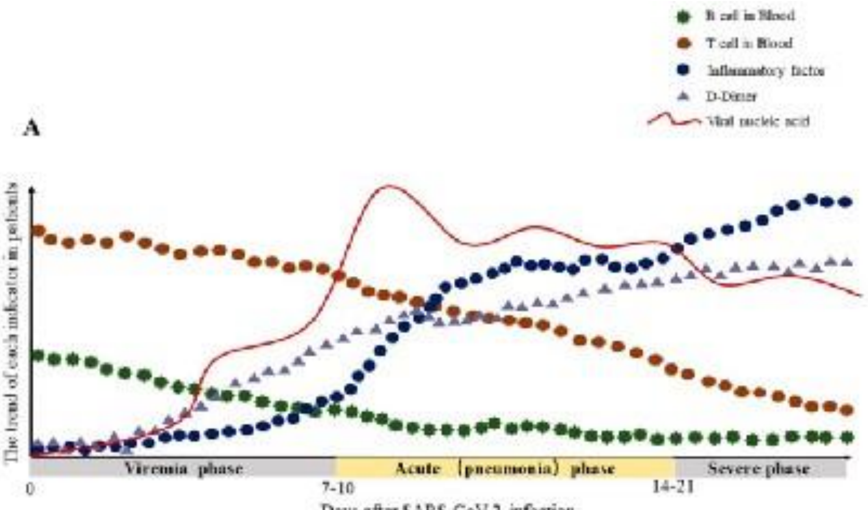

1.5.4. Selection of endpoints

The selection of those endpoints based on the objectivity and to present the most reliable to assess moderate to severe infection. It is expected that the majority of the cases would be mild. Therefore, the markers used (seven-category ordinal scale), which capture the requirement of hospitalization and Oxygen requirement, reflect the best assessment for such a spectrum.

1.5.5. Benefit/Risk Assessment

1.5.5.1 Known Potential Risks:

- Favipiravir use has been associated with adverse drug reactions including increase of uric acid serum level (~4.8%), diarrhea (~4.8%), decrease neutrophil count (1.8%), and increase in AST and ALT (1.8%, 1.6%) respectively.
- Hydroxychloroquine use has been associated with adverse drug reactions including bone marrow suppression (anemia, leukopenia, agranulocytosis, and thrombocytopenia), QTc prolongation, irreversible retinopathy with retinal pigmentation changes and visual disturbances, nausea, vomiting, diarrhea, and abdominal pain, and skin rash

1.5.5.2 Known Potential Benefits:

- Favipiravir has shown shorter viral clearance time when compared to lopinavir/ritonavir in a study of 80 patients who has Covid-19 (4 days versus 11 days, P < 0.001). The Favipiravir arm also showed significant improvement in chest imaging compared with the lopinavir/ritonavir arm, with an improvement rate of 91.43% versus 62.22% (P = 0.004)[5].
- Hydroxychloroquine use associated with shorter time to clinical recovery manifested by body temperature recovery and cough remission compared to standard of care group. Improvement in chest CT also has been observed in HCQ group

1.5.5.3 Assessment of Potential Risks and Benefits

The WHO declared COVID-19 as a pandemic on March 12th, 2020 [1]. The transmission dynamics, disease progression and severity of COVID-19 among human being is under investigation yet to be understood. Looking at the dramatic increase of confirmed cases of COVID-19 in Saudi Arabia and worldwide necessitate exploring therapeutic options that showed promising killing activity against COVID-19 in vitro and have relative safety profiles. Hydroxychloroquine has been used for long time for patients with autoimmune disease. It is well known of its high tolerability profile. Favipiravir has been used as anti-influenza drug for years with good clinical outcomes and have a relative safe profile

2. STUDY OBJECTIVES AND ENDPOINTS

2.1 Primary Objectives

The improvement of two points (from the status at randomization) on a seven-category ordinal scale* or live discharge from the hospital, whichever came first.

*The seven-category ordinal scale consists of the following categories:

8. Not hospitalized with resumption of normal activities
9. Not hospitalized, but unable to resume normal activities
10. Hospitalization, not requiring supplemental oxygen
11. Hospitalization, requiring supplemental oxygen
12. Hospitalization, requiring nasal high-flow oxygen therapy and/or non-invasive mechanical ventilation
13. Hospitalization, requiring ECMO and/or invasive mechanical ventilation
14. Death.

2.2 Secondary Objectives

1. To evaluate the progress in clinical status of COVID-19 patients in both arms
2. To monitor the viral shedding duration and PCR test conversion days from positive to negative.
3. Evaluate the safety of investigational therapeutics as compared to the control arm.
4. To evaluate difference in the complications and prognosis of COVID-19 patients between the two groups.

2.3 Primary Endpoint

The primary endpoint is the time to clinical improvement, defined as the time from randomization to an improvement of two points (from the status at randomization) on a seven-categoryordinal scale or live discharge from the hospital, whichever came first.

2.4 Secondary Endpoint

1. Clinical status assessed by the seven-category ordinal scale.
2. The requirement of ICU admission or Mechanical ventilation
3. 28- and 90-days mortality.
4. PCR test negative conversion rate and days from positive to negative**
5. Evaluate the safety of investigational therapeutics as compared to the control arm.
6. Length of hospital stay.
7. Duration of fever.
8. QT prolongation (Daily follow up during the concomitant use of both medications and regularly till Day14)

2.5 Safety Outcome Measures:

Safety assessments will consist of monitoring and recording all adverse events and serious adverse events occurring during the regular monitoring of hematology and blood chemistry, ECG, regular measurement of vital signs, performance of physical and neurological examinations if needed.

3. STUDY DESIGN

This is an Open label, multicenter, randomized controlled clinical trial to evaluate the safety and efficacy of novel therapeutic agents in hospitalized adults diagnosed with COVID-19. It is a multicenter trial that will compare Favipiravir plus Hydroxychloroquine combination (experimental arm) to a control arm.

4. STUDY POPULATION

4.1 Inclusion Criteria

1. Should be at least 18 years of age,
2. Male or nonpregnant female,
3. Diagnosed with COVID-19 by PCR confirmed SARS-coV-2 viral infection.
4. Able to sign the consent form and agree to clinical samples collection (or their legal surrogates if subjects are or become unable to make informed decisions)..
5. Moderate or Severe COVID-19, defined as oxygen saturation (Sao2) of 94% or less while they were breathing ambient air or significant clinical symptoms that require hospital admission.
6. Patients had to be enrolled within 10 days of disease onset.
7. Not part of any other clinical trial and agrees not to participate in any.

4.2 Exclusion Criteria

1. Patients who are pregnant or breastfeeding.
2. Will be transferred to a non-study site hospital or discharge from hospital within 72 hours.
3. Known sensitivity/allergy to hydroxychloroquine or Favipiravir
4. Current use of hydroxychloroquine for another indication
5. Prior diagnosis of retinopathy
6. Prior diagnosis of glucose-6-phosphate dehydrogenase (G6PD) deficiency
7. Major comorbidities increasing the risk of study drug including: i. Hematologic malignancy, ii. Advanced (stage 4-5) chronic kidney disease or dialysis therapy, iii. Known history of ventricular arrhythmias, iv. Current use of drugs that prolong the QT interval, Severe liver damage (Child-Pugh score ≥ C, AST> 5 times the upper limit), HIV.
8. The investigator believes that participating in the trial is not in the best interests of the patient, or the investigator considers unsuitable for enrollment (such as unpredictable risks or subject compliance issues).
9. Clinical prognostic non-survival, palliative care, or in deep coma and no have response to supportive treatment within three hours of admission
10. Patient with irregular rhythm or the QTc (corrected QT interval) in the baseline ECG more than 490ms.

11 Patient with a history of heart attack (myocardial infarction)

12 Patient with a family history of sudden death from heart attack before the age of 50

13 Take other drugs that can cause prolonged QT interval

14 Patient who is receiving immunosuppressive therapy (cyclosporin) which cannot be switched to another agent or adjusted while using the investigational drug

15 Gout/history of Gout or hereditary xanthinuria or xanthine calculi of the urinary tract.

16 Serum uric acid level > 2 times upper level of normal associated with symptoms of gout

4.3 Patient recruitment

In the participating centers, daily notification from the microbiology laboratory for the new positive cases to the research coordinators. Then assessment of the eligibility would be done.

5. PREMATURE DISCONTINUATION OF THE STUDY

Premature discontinuation of the trial would be based on the decision of DSMB or the investigator initiated based on the following:

- Adverse event: clinical or laboratory event that in the medical judgment of the investigator, for the best interest of the patient are grounds for discontinuation
- Major deviation from the protocol: the patient's findings or conduct failed to adhere to the protocol requirements.
- Other reason: e.g., administrative problem such as termination of study by the sponsor.

6. TREATMENTS

7.1 Description of investigational drug and reference therapy

7.2 Packaging and labelling[13,14]

Table 2 Packaging and labelling of the study medications

**Generic Name**	**Favipiravir**	**Hydroxychloroquine**
**Trade Name**	Avigan®	Plaquenil®
**Dosage Form**	Tablet	- Tablet<br>- Suspension (extemporaneous preparation) manufactured in house
**Tablet Strength**	200 mg	- 200 mg<br>- 25 mg/mL for suspension formulation
**Appearance and Packaging**	Light-yellow, film-coated round tablet<br>Boxes of 100 tablets in press through packaging	White to off white, film coated tablet<br>Bottle of 100 tablets
**Manufacturer**	FujiFilm Toyama Chemical Co.	Concordia Pharmaceuticals Inc
**Supplier**	Satellite pharmacy	Satellite pharmacy

-Both drugs are available in oral dosage form (oral tablet). Hydroxychloroquine is also available as suspension that is manufactured at King Abdulaziz Medical City outpatient compounding pharmacy.

Reference Therapy:

Standard of care is defined as: Treatment that is accepted by medical experts as a proper treatment for Covid-19 disease. Standard care comprised, as necessary, supplemental oxygen,

noninvasive and invasive ventilation, antibiotic agents, vasopressor support, renal-replacement therapy, extracorporeal membrane oxygenation (ECMO), and antiviral therapy except Favipiravir. Also, it may include intravenous fluids and medications for symptoms relieve.

Although it would not be preferred to use medication that has antiviral activity, the treating physician would have the authority/ autonomy to start the patient on antiviral but not Favipiravir, i.e., Lopinavir–Ritonavir, interferon, ribavirin, Hydroxychloroquine or Remdesivir). Anyone of those medications with possible antiviral activity against SARS-CoV-2 would be accepted to be used in the case that treating physicians thought it would be the best of interest for the patient.

7.3 Treatment assignment

In the participating site, the research coordinator/ principal investigator for the site will check all positive reported COVID-19 by PCR confirmed SARS-coV-2 viral infection. Then an assessment of the eligibility by the research coordinator and assessing inclusion/exclusion criteria. The possible study participant can be assessed in the first 72 hours of admission regarding eligibility. Once eligible, informed consent will be obtained. Randomization will be done in 1:1 whether to intervention or control arm and stratified by the center.

7.4 Randomization

Eligible participants will be randomized in a 1:1 ratio to either the combination group (Favipiravir and Hydroxychloroquine) or a control group. Randomization will be stratified by clinical site.

The patients will be randomized utilizing Web based data entry System. The sequence of treatment assignment will be determined prior to the start of the study.

7.5 Storage conditions

Table 3 Storage Conditions for study medications

**Drug**	**Favipiravir**	**Hydroxycholoroquine**

**Storage Condition**	Storage at room temperature [20° to 25°C (68° to 77°F)]	Store at room temperature [20° to 25°C (68° to 77°F), allows excursions between 15° and 30°C (59° and 86°F)].
**Stability**	Must be dispensed in a tight container	Must be dispensed in a tight, light-resistant container to keep ingredients active until expiry date

7.6 Treatment compliance

Compliance with the study drug will be assessed by the study coordinator at each study visit and he / she will be required to record in the CRF any missed dose, the reason for missing doses, any adverse effect, and any associated issues, beginning from visit 1.

7.7 Accountability and Destruction

In compliance with the International Conference on Harmonization (ICH) guidelines, Title E6-Good Clinical Practices section 4.6, and CFR 21 Part 312.61, the Investigator is responsible for maintaining records reflecting the receipt and dispensation of the investigational product. Additionally, the Investigator must comply with any local regulations regarding the proper documentation of study drug accountability.

The study drug must be stored and handled in accordance with the Sponsor's instructions.

At the end of the study, remaining therapeutic unit will be destroyed locally and a certificate of destruction will be issued by King Abdulaziz Medical City.

7.8 Concomitant treatments

B. Patient will remain on medications he/she is taking prior to the study. However Use Favipiravir must be with caution while using acetaminophen aution while usingns he/she is taking prior than 3000mg/day (or less in patients with hepatic insufficiency)

7. PROCEDURES AND ASSESSMENTS

The Study comprises of three major parts Screening, Treatment and Follow-up period. Each Part consists of specified procedures to be done and assessments to be carried. All study procedures will be conducted in King Abdul-Aziz Medical City National Guard Health Affairs in Riyadh. The investigator and supporting study team will be responsible to document all the procedures and assessments done in the appropriate source document and the patient e CRFs. All procedures and assessments will support the safety and validity of conclusions drawn from the study protocol. Procedures and assessments such as vital signs, laboratory tests…etc will follow in-house policies and guidelines. When multiple assessments are taken at the same time point, the most out-of-range value shall be considered

7.1 Screening/Baseline

In the participating site, the research coordinator/ delegated study personnel for the site will check all positive reported COVID-19 by PCR confirmed SARS-coV-2 viral infection. Then an assessment of the eligibility by the research coordinator and assessing inclusion/exclusion criteria. The possible study participant can be assessed in the first 72 hours of admission regarding eligibility. Once eligible, informed consent will be obtained. Randomization will be done in 1:1 whether to intervention or control arm and stratified by the center.
Daily assessment and data reporting will be done by the research coordinator/ delegated study personnel including the side effect using the included CRF.

While the management will be done by the treating physician and even stopping the medication in case of concerns. Stopping the interventional medication need to be justified; where a form would be filled.

7.2 Treatment Period

The treatment intervention would be for a maximum of 10 days from randomization and it would be as follow:

Favipiravir for 10 days as follow:

Administer 1800 mg (9 tablets) by mouth twice daily for one day, followed by 800mg (4 tablets) twice daily (total days of therapy is 10 days)

Hydroxychloroquine for 5 days:

(400mg) twice daily on day 1; for days 2-5 (200mg) twice daily.

7.3 Safety monitoring

Site PI should follow on a daily basis all the possible AEs while using the investigational medications and up to day-14. This includes regular monitoring of hematology and blood chemistry. Daily ECG and QT interval monitoring. And LFT every other day. In case of any concerns that might require holding/stopping medication, causality assessment should be done by PI and with a consultation to an independent clinician.

Details of cardiac toxicity monitoring as follow:

1. A baseline ECG before starting then daily ECG after.

2. Daily ECG to be done through day 1-10 or twice daily if the corrected QT interval more than 480ms.

2. Maintain serum level of Mg>1.0 meq, and K>4.5 meq throughout the 10 days of therapy.

3. If the QTc prolonged more than 500ms after starting, stop the medication.

4. Avoid any other QT prolong medication during the regimen.

5. If the patient develops torsades de-pointes, stop the medication immediately, follow ACLS protocol and call the cardiologist on-call.

If you have any query or help, please call for cardiology team in you center.

7.4 Follow-up Period

The follow up period would be till discharge from the hospital or day 28 from randomization.

Where serial oropharyngeal swab or lower respiratory samples will be obtained on day 1 (-3 days) (before therapy was administered) and on days 5±1 day, 10±2 days, 14±2 days, 21±2 days, and 28±3 days or until 2 consecutive testing are negative which comes first. Any extra Respiratory PCR COVID19 samples requested by the treating team will be recorded. Other laboratory investigations will be recorded as per flow chart.

7.5 Laboratory Method

The laboratory tests that intended to be used for upper (Bronchoalveolar lavage, tracheal aspirate, and Sputum) and lower (Nasopharyngeal swab and oropharyngeal swab) respiratory samples should follow the updated national guidelines. BAL and tracheal aspirate should be 2-3 mL in a sterile, leak-proof, screw-cap container while Sputum collected directly into a sterile, leak-proof, screw-cap container. For the collection of Nasopharyngeal and oropharyngeal, synthetic fiber swabs with plastic shafts should be used. It is not recommended to use swabs with wooden shafts or calcium alginate swabs, which might have a negative impact on PCR results. Swabs should be immediately placed (separate or combined) into a sterile tube containing 2-3 ml of viral transport media (VTM).

Store samples at 2-8°C and ship on an ice pack to the designated laboratory. Samples can be stored at 2-8°C for ≤48 hours; if longer storage is needed, samples should be stored at –70 °C. If the sample is frozen at -70°C, ship on dry ice.

Testing will be limited to qualified laboratories with a certified Biological Safety Cabinet Class II (BSC-II) in a Biosafety level 2 facility (BSL-2) with negative pressure room and those approved by Saudi CDC. Diagnostic RT-PCR will be performed by each hospital's laboratory or the reference/National laboratory of the Saudi Ministry of Health. Additionally, respiratory and blood samples might be sent to King Abdullah International Research Center for further testing. The testing procedure includes extracting ribonucleic acid (RNA) from respiratory specimens using the MagNA Pure 96 Viral NA Kit. The extracted nucleic acids will be tested by rRT-PCR

targeting the upstream SARS-CoV-2 envelope protein gene. The laboratory should follow the Saudi CDC recommendations for Results interpretation of Real-Time RT-PCR and follow the manufacturer recommendations for the defined cut-off CT value for the positive results based on the CT value for each target gene.

8. FLOW CHART

Table 4: Flow Chart

	Study period											
	Baseline	Post allocation										Closeout
Timepoint study days	D0	D1	D2	D’’	D5	D’’	D9	D10	D’’	D14	D21	D28
Enrolment and assignment												
Eligibility assessment	X	X										
Informed consent	X	X										
Randomization	X	X										
Baseline data	X	X										
Study drug administration												
Favipiravir		X	X	X	X	X	X	X				
Hydroxychloroquine		X	X	X	X							
Adverse effect reaction		X	X	X	X	X	X	X	X	X		
Serious adverse event assessment		X	X	X	X	X	X	X	X	X	X	X
Clinical data collection												
Seven-category ordinal scale and CRF	X	X	X	X	X	X	X	X	X	X	X	X
Laboratory data collection												
Covid-19 PCR from Respiratory sample *	X	X			X			X		X	X	X
CBC, renal profile and LFT on **Study Drug**	X	X	X	X	X	X	X	X		X	X	
CBC, renal profile and LFT on **SoC**	X	X			X			X			X	

ECG for patient on **Study Drug**	X	X	X	X	X			X				
ECG for patient on **SoC**	X	X			X			X				

9 STATISTICAL CONSIDERATIONS

9.1 General Considerations

This is a randomized, Open-Label trial comparing Favipiravir tables + Hydroxchloroquine tablets standard of care group for the treatment of subjects with moderate to severe SARS-COV-2 infection. The Intention to treat (ITT) analysis will include all subjects randomized. The primary analysis population for the evaluation of both efficacy and safety outcomes will be a modified ITT population, and include all subjects who have been randomized and in which the study drug (Favipiravir tables + Hydroxchloroquine or Standard of care) was started and the patient did not withdraw consent.

9.2 Sample Size and Power Considerations

9.2.1 **Assumptions and Study Hypothesis:**

a. **Time to discharge or clinical improvement by 2 points** in the standard of care arm is expected to be (median: 11 days; Cai Q, Y.M., Liu D, Chen J, Shu D, Xia J, , Experimental Treatment with Favipiravir for COVID-19: An Open-Label Control Study. 2020.).

b. The exact treatment effect from Favipiravir tables + Hydroxchloroquine is not exactly known but can be approximated using prior clinical studies. A study comparing the effect of Favipiravir to lopinavir/ritonavir on virus clearance has shown a 64% reduction in time to viral clearance in the Favipiravir arm. This was equivalent to a median of 11 days. We assume that clinical improvement might take place a little later to viral clearance and therefore we assume that the treatment arm might result in a minimum clinically meaningful expected reduction of 30% (Median 7.7 days). This is equivalent to a Hazard ratio of 0.7.

c. We further assume, that 50% of the patients in the control group will have clinical improvement/ discharged and 70% will have clinical improvement/discharged in the treatment arm

d. It is anticipated that very few of these subjects will be randomized and not start study treatment (and so be excluded from the primary analysis) or be lost to follow-up (and so have missing data for the primary endpoint). Given certain uncertainties however, we have included a nominal 10% drop out rate.

e. The primary hypothesis of the current study is H0: HR =1 vs. H1: HR ≠1 ; and HR is the hazard ratio of treatment compared to control arm.

9.2.2 **Sample Size Estimation for Classical Two Arm Parallel Design:**

Under the classical two arms parallel design the total effective sample sizes needed is 472 subjects (236 subjects per group). The Estimated sample size achieve 85 % power to detect a minimum of 30% reduction in the median time, to clinical improvement by at least two points or hospital discharge, in the treatment group compared to the control group. The median time in the treatment arm is 11 days under the null hypothesis and 7.7 days under the alternative hypothesis. The median time in control group under the null hypothesis is 11 days. The two survival curves will be analyzed using one sided cox proportional hazard regression. The significance level of the test is 0.025. Assuming 10% drop out rate, the overall sample size for the trial is estimated to be 520 (260 per group). Sample size adjustment will be considered after interim analysis of 60% of the subjects' recruitment and based on DSMB recommendation.

Sensitivity analysis

We have performed several sensitivity analyses to determine the effect of different assumptions on the sample size. Figure 2A, shows the relationship between the proportion of event in the treatment (Prev2), the proportion of event in the control (Prev1) and the power of the study. Increase in the events in both groups lead to increase in the overall study power up to 90%. Simultaneous decrease in the events could lead less of power to as low as 78%. Reduction of events in the treatment group alone does not lead to loss of power below the accepted norm of 80%. Because reduction in the proportion of events in treatment group has the largest impact, we examined the impact of overestimation of the HR and the proportion of events in the treatment group on power. Figure 2B, shows that regardless of the number of events a slight change in the HR than the assumed will have severe impact on power. However, the current design could accommodate an overestimation of HR by only 10% (.27 instead .3) before power falls under desired level.

A. Impact of proportion of events in treatment and control on Power.

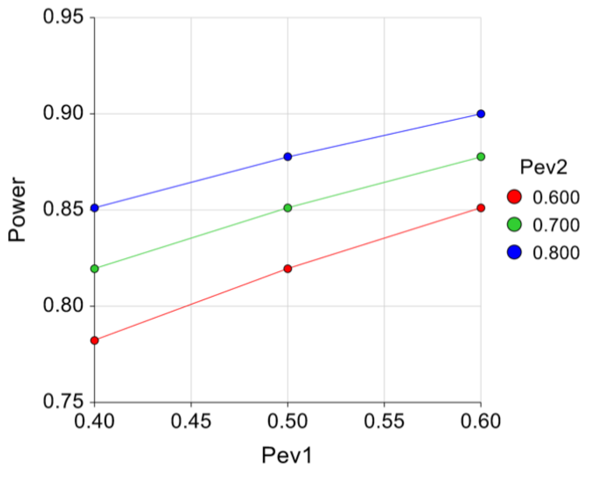

B. Impact of HR and proportion of events in treatment arm on power

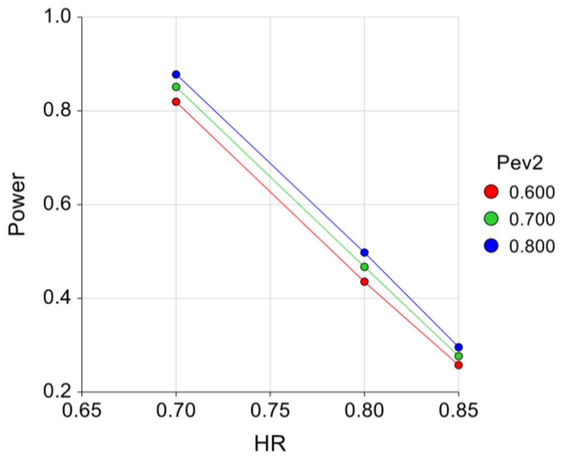

Figure 2 . A: Impact of proportion of events in treatment and control on Power. B: Impact of HR and proportion of events in treatment arm on power.

9.3 Study Interim Analysis

The current study will have a single interim analysis which will take place at the completion of the recruitment and follow-up of 60% of the total number of subjects (i.e. 312). The interim analysis is designed to test for early stopping for futility or efficacy as well as for sample size re-estimation. The interim analysis and final analysis will be based on the method of the sum of the stage wise p-value discussed in Mark and Chang, 2008. The table below, describe the interim analysis testing boundaries.

Boundary	Decision
Alpha1 = 0.01	Stop the trial for early efficacy if the interim analysis p-value is less than 0.01
Beta1 =0.25	Stop the trial for futility if the interim analysis P-value is equal to or larger than 0.25
Alpha2=0.1832	Declare the trial significant if the sum of the interim analysis and final stage P-values are less than 0.1832

The sample size Re-estimation will be based on the ratio of the planned effect size to the observed effect size from the interim analysis according to the following formula:

$$N = \left(\frac{E_0}{E}\right)^a N_0$$

Where a is a constant which will be set to 2 and N_0 is a number chosen to be slightly larger than the classical sample size per group, E_0 is the planned effect size of .7 and E is the observed effect size from the interim analysis.

9.4 Statistical Analysis

A detailed statistical analysis plan will be developed before undertaking any comparative analyses of outcomes. The following provides a brief summary of approach to analysis for the primary endpoint.

Analysis of the primary endpoint:

The primary endpoint of the current study is Median time to clinical improvement by at least 2 points or discharge from the hospital. The number and percent of subjects who met the endpoint by day 28 of follow up will be calculated and tabulated. Kaplan-Meier plot of time from randomization to endpoint will be generated (censoring at 28 days if completed follow-up, or at date of last contact if lost to follow-up prior to 28 days), with associated table showing: number censored and number (%) meeting the endpoint, median time to endpoint and associated HR with 95% confidence interval; p-value from cox proportional hazard regression.

For secondary endpoints:

- Quantitative variables such as duration of ICU stay and change from baseline in clinical scores, etc., are expected to have reasonably skewed distributions and may be subject to censoring (e.g., for subjects in hospital on Day 28; these will be compared between randomized arms using non-parametric tests (e.g., Wilcoxon's Test adapted, if necessary, to handle censoring).
- Analysis of the ordinal scale endpoints will use a proportional odds model with an indicator variable for randomized treatment. The Wald test will be used to generate a p-value comparing treatments, as well as the estimated proportional odds ratio comparing treatments with associated 95% CI.

- Analysis of AE data will primarily be descriptive based on MedDRA coding of events. The proportion of subjects experiencing an SAE and the proportion experiencing a Grade 3 or higher AE will be compared between randomized arms using Fisher's Exact Test.

For enrolled subjects who were not randomized (i.e. screen failures) or who were randomized but did not receive the treatment, the final analysis will detail safety (deaths and SAEs), and reasons they were not randomized or did not received treatment respectively.

10. ADVERSE EVENTS

10.1Definitions

Adverse Event (AE). According to International Conference on Harmonization (ICH) E2A, an adverse event (or adverse experience) is any untoward medical occurrence in a subject or clinical investigation subject administered a pharmaceutical product and which does not necessarily have to have a causal relationship with this treatment. An AE can therefore be any unfavourable and unintended sign (including an abnormal laboratory finding, for example), symptom, or disease temporally associated with the use of a medicinal product, whether or not considered related to the medicinal product.

Serious Adverse Event (SAE). A serious adverse event (SAE) is any untoward medical occurrence that, at any dose:

- Results in death
- Is life-threatening, (Note: The term "life-threatening" in the definition of "serious" refers to an event in which the subject was at risk of death at the time of the event. It does not refer to an event, which hypothetically might have caused death if more severe.)
- Requires hospitalization or prolongation of existing hospitalization
- Results in disability/incapacity or
- Is a congenital anomaly/birth defect.

Medical or scientific judgment should be exercised in deciding whether reporting is appropriate in other situations, such as important medical events that may not be immediately life threatening or result in death or hospitalization but may jeopardize the subject or may require medical or surgical intervention to prevent one of other outcomes listed in the above definition. These should also be considered serious.

Examples of such events are invasive or malignant cancers, intensive treatment in emergency room or at home for allergic bronchospasm, blood dyscrasias or convulsions that do not result in hospitalization, or development of drug dependency or drug abuse.

For the purpose of this trial, the information related to the emergency visits due to hyperammonemia will be collected specifically on the CRF, and should not be consider an AE or SAE.

10.2 Adverse event with interventional medication

Favipiravir Adverse Drug Reactions

- Major adverse drug reactions include increase of uric acid serum level (~4.8%), diarrhea (~4.8%), decrease neutrophil count (1.8%), and increase in AST and ALT (1.8%, 1.6%) respectively.
- Other clinically significant ADRs include skin rash, neurological and psychiatric symptoms (e.g. suddenly running away, wandering around), anaphylactic shock, pneumonia, hepatic dysfunction, acute renal failure, decrease in white blood cell count and platelet count, and hemorrhagic colitis.

HYDROXYCHLOROQUINE ADVERSE DRUG REACTIONS

- Adverse drug reactions have been reported after administering hydroxychloroquine include:

- Blood disorders: Bone marrow suppression (anemia, leukopenia, agranulocytosis, and thrombocytopenia)
- Cardiac disorders: QTc prolongation
- Ear disorders: Vertigo, tinnitus, and nystagmus
- Eye disorders: Irreversible retinopathy with retinal pigmentation changes and visual disturbances
- Gastrointestinal disorders: Nausea, vomiting, diarrhea, and abdominal pain
- Hepatobiliary disorders: Liver function tests abnormal and acute hepatic failure
- Metabolism and nutrition disorders: Decreased appetite, hypoglycemia, porphyria, weight decreased
- Nervous system disorders: Headache, dizziness, seizure, ataxia and extrapyramidal disorders such as dystonia
- Psychiatric disorders: Affect/emotional lability, nervousness, irritability, nightmares, psychosis, and suicidal behavior
- Skin and subcutaneous tissue disorders: Rash, pruritus, pigmentation disorders in skin and mucous membranes, hair color changes, alopecia.

10.3Handling of Non-Serious Adverse Events

All AEs encountered during the clinical study will be reported on the CRF. Adverse events, whether or not associated with study medication administration, will be recorded on the Adverse Event form of the CRF.

The information to be entered in the CRF will include:

- The time of onset of any AE or the worsening of a previously observed AE
- The specific type of reaction in standard medical terminology
- The duration of the AE (start and stop dates)
- The severity of the adverse event (AE). The severity should be rated as:

    - Mild: discomfort noted, but no disruption of normal daily activity.
    - Moderate: discomfort noted of sufficient severity to reduce or adversely affect normal activity.
    - Severe: incapacitating, with inability to work or perform normal daily activity.
- An assessment of the relationship of the adverse event (AE) to the study medication, i.e., according to the definitions below:
    - Related: with a reasonable causal relationship to the investigational product
    - Not Related: without a reasonable causal relationship to the investigational product
    - Other: in such case, investigator's causality assessment should be clearly specified.
- Description of action taken in treating the AE and/or change in study medication administration or dose.

As far as possible, all investigators should follow-up participants with AEs until the event is resolved or until, in the opinion of the investigator, the event is stabilized or determined to be chronic. Details of AE resolution must be documented in the CRF. Any significant changes in AEs should be reported even though the subject has completed the study, including the protocol-required post-treatment follow-up.

10.4Handling of Serious Adverse Events

All adverse drug events encountered during the clinical study will be reported on the CRF. Serious adverse events (SAEs), whether or not associated with study medication administration, will be recorded both on the Adverse Event form of the CRF as for non-serious AE and the SAE form.

ALL SAEs occurring during this study and up to 30 days after a subject discontinued or completed the study, whether or not related to the administration of study medication, must be reported by faxing the completed SAE form, after reviewing the report for consistency and accuracy within 24 hours of awareness by the investigator, to KAIMRC / IRB.

As far as possible, investigators should follow-up participants with AEs until the event is resolved or until, in the opinion of the investigator, the event is stabilized or determined to be chronic. Details of AE resolution must be documented in the CRF. Participants should be followed-up for 30 days after receiving the last dose of study medication and any AEs, which occur during this time, should be reported according to the procedures outlined above. Any significant changes in AEs should be reported even though the subject has completed the study, including the protocol-required post-treatment follow-up.

10.5 Regulatory Responsibility

The sponsor has a responsibility to report to the regulatory authorities and Ethics Committee safety information according to the local regulations. Therefore, prompt notification of serious adverse events by the investigator is required so that reporting timelines can be met and also to ensure ethical responsibilities towards the safety of other subjects are met.

The sponsor will also inform all participating investigators and Ethics Committee according to the local regulations.

11. ETHICAL AND REGULATORY STANDARDS

11.1 Ethics and Good Clinical Practices

This study must be carried out in compliance with the protocol and in accordance with the laws and regulations of Saudi Arabia, and the sponsor or their representative's standard operating procedures. These are designed to ensure adherence to Good Clinical Practice, as described in the following documents:

ICH Harmonized Tripartite Guidelines for Good Clinical Practice 1996.

- Declaration of Helsinki, concerning medical research in humans (Recommendations Guiding Physicians in Biomedical Research Involving Human Subjects, Helsinki 1964, amended Tokyo 1975, Venice 1983, Hong Kong 1989, Somerset West 1996, Edinburgh, Scotland, October 2000, Washington 2002, Tokyo 2004, Seoul, October 2008) Brazil, October 2013.

The Investigator agrees, when signing the protocol, to adhere to the instructions and procedures described in it and thereby to adhere to the principles of Good Clinical Practice that it conforms to. A copy of the Declaration of Helsinki is provided in the Investigator study file at each site.

11.2 Ethics Committee

Before implementing this study, the protocol, the proposed informed consent form and other information to subjects must be reviewed by an appropriate Institutional Review Board/Independent Ethics Committee (IRB/IEC). A signed and dated statement that the protocol and informed consent have been approved by the IRB/IEC must be given to the Sponsor before study commencement. The name and occupation of the chairman and the members of the IRB/IEC must be supplied to the Sponsor. Any amendments to the protocol, which need formal approval as required by local law, must be approved by this committee. The IRB may be notified of all other amendments (i.e. administrative changes).

The study may not start before written approval has been obtained for the protocol and the informed consent form.

11.3 Informed consent

In obtaining and documenting informed consent, the investigator should comply with the applicable regulatory requirement(s), and should adhere to GCP and to the ethical principles. Prior to the beginning of the trial, the investigator should have the IRB written approval of the written informed consent form and consenting process.

The investigator, or a person designated by the investigator, should fully inform the subject, subjects family or the subject's Legally Acceptable Representative (LAR), of all pertinent aspects of the trial including the nature of the study, its purpose, the procedures involved, the expected duration, the potential risks and benefits involved and any discomfort it may entails. All questions about the trial should be answered to the satisfaction of the subject or the subject's family member or subject's LAR.

Due to the nature of the study and the risk of spreading the virus to the study team, and after waiver is granted by the IRB, special measures related to the consenting and the documentation process will be implemented. These measures are:

- Prior to a subject's participation in the trial, the written informed consent form should be signed and personally dated by the subject and the PI and/or the delegated person who explained the trial to the patient, using two original copies, one to be given to the patient and one with the study team. . To reduce the risk of spreading the virus, the original consent forms will be kept in the secure place with minimal access in the hospital before filing.
- If the subject is unable to give the consent, a subject's family member or subject's LAR will give the consent. In some situations where the family or subject's LAR are not able to be present, an oral consent over the phone will be obtained in a timely manner followed by sending the consent form to be signed and collected via the email communication or mobile phone if possible at the earliest opportunity valuable or to be signed at a later time at the site when allowed.
- If a subject is unable to read or if a family member or subject's LAR is unable to read, then an impartial witness should present during the entire informed consent discussion, and signs the consent form in addition to the subject or the subject's family member or subject's LAR.
- Subjects who are unable to read and who do not speak Arabic nor English as their first language have the consent form read to them by a qualified interpreter and that the interpreter signs the consent form as well as the subject, subject's family member, subject's LAR and the PI.
- If the subject is unable to give a consent and has no family member or subject's LAR or in case the PI or the study team could not reach to a family member or subject's LAR after doing all possible efforts, the PI has to seek the consent from the subject's attending physician (most responsible physician) for the enrollment in the trial.

- Procedures to inform, at the earliest feasible opportunity, the subject (i.e., if his/her condition improves), a family member, a subject's LAR of the subject's inclusion in the trial, the details of the research, and the right to discontinue the subject's participation at any time without penalty or a loss of entitled benefits.

Any changes to the proposed consent form suggested by the investigator must be agreed to by the Sponsor before submission to the IRB/IEC and a copy of the approved version must be provided to the Sponsor after IRB/IEC approval.

12. STUDY MONITORING, DATAMANAGEMENT AND QUALITY ASSURANCE

As an Investigator Initiated Trial, this study will not be formally monitored. A research assistant who has expertise in data entry will enter data into a password-protected database. Data will be entered and double checked for accuracy. After resolution of any discrepancies and a combination of manual and automated data- review procedures, the final data set will be subject to a quality assurance audit.

To ensure the quality of the clinical data across all participants and sites, a clinical data management review will be performed on all subject data. During this review, subject data will be checked for consistency, omissions and any apparent discrepancies. In addition, the data will be reviewed for adherence to protocol. To resolve any questions arising from the clinical data review process, data queries will be sent to the site for completion.

12.1Case Report Forms (CRFs) & Recording of Data

It is the responsibility of the Investigators to record all observations and other data pertinent to the clinical investigation. For this study an electronic CRF (eCRF) will be used.

Data on subjects during the trial will be documented in an anonymous fashion and the subject will only be identified by the subject number, and his/her initials. The Investigator must maintain source documents for each patient in the study. All information in the study database must be traceable to these source documents, which are generally maintained in the patient's file. The source documents should contain all demographic and medical information, including laboratory data, and a copy of the signed informed consent form, which should indicate the study number and title of the trial.

Essential documents, as listed below, must be retained by the Investigators for as long as needed to comply with national and international regulations. The Investigators agree to adhere to the document retention procedures by signing the protocol.

Essential documents include:

- IRB/IEC approvals for the study protocol and all amendments;
- Source documents and laboratory records;
- Patients' informed consent forms;

- All other pertinent study documents.

12.2Data Safety Monitoring Board

A Data and Safety Management Board (DSMB) will be convened to monitor the unblinded data from the trial focusing mainly on the assuring that the study is following the protocol properly and to monitor the safety issues related to the trial. This committee will be composed of independent team members with relevant therapeutic and/or biostatistical experience to allow for the ongoing review of data from this trial. The DSMB will meet regularly throughout the course of the trial and when AEs trigger study pausing/stopping criteria are triggered. Further details will be described in an DSMB charter prior to the start of the clinical trial.

13. ADMINISTRATIVE RULES

13.1 Secrecy Agreement

By signing the protocol, the investigator agrees to keep all information provided by the sponsor in strict confidence and to request similar confidentiality from his/her staff and the IRB/EC. Study documents provided by the sponsor will be stored appropriately to ensure their confidentiality.

The information provided by the sponsor or the partner to the investigator may not be disclosed to others without direct written authorization from the sponsor/ partner, except to the extent necessary to obtain informed consent from patients who wish to participate in the trial.

13.2 Protocol amendments

Any change or addition to this protocol requires a written amendment that must be approved by the sponsor and the investigators.

Before implementation, the investigators must transmit all major amendments to the Ethics Committees having examined the initial protocol. The investigators must transmit a copy of the Ethics Committee's opinion to the sponsor.

All minor amendments must be notified by the investigators to the Ethics Committee having examined the initial protocol.

13.3 Record retention in investigation centre(s)

The investigator must maintain all study records, subject files and other source data for at least 15 years.

13.4 Insurance compensation

The sponsor certifies having taken out a liability insurance policy which covers the investigators and his co-workers and which is in accordance with the local laws and requirements.

13.5 Sponsor audits and inspections by regulatory agencies

The investigator should be informed that an audit may be carried out, at the request of the sponsor during or after the end of the study.

The investigator should be informed that the Saudi Food & Drugs Authority, or other Regulatory Authorities may also carry out an inspection.

The investigator must allow representatives of the Regulatory Authorities and persons responsible for the audit:

- to inspect the site, facilities and material used for the study,
- to meet the members of his team involved in the study,
- to have access to study data and source documents,
- to consult all of the documents relevant to the study.

13.6 Discontinuation of the study

In case of premature discontinuation of the study, the sponsor will inform in writing, all investigators, Ethics Committees and Regulatory Authorities about the reasons for cancelling the study.

14. TIMELINES

IRB Approval ………………………………………………………… April 2020

Saudi Food & Drugs Authority Approval ………………… May 2020

First Site Initiation Visit ………………………………… May 2020

First Patient First Visit ………………………………… May 2020
Last Patient First Visit …………….. May 2021
Last Patient Last Visit ………….. Nov 2021
Database lock ……………………………………… Nov 2021
Final Study Report …………………………………. Dec 2021

15. PUBLICATION

The intention is to publish the results of the complete study at conclusion. All information obtained during the conduct of this study will be regarded as confidential and written permission from the Sponsor is required prior to disclosing any information relative to this study. A formal publication of data collected as a result of the study is planned and will be considered a joint publication by all Investigators and the appropriate Sponsor personnel. Authorship will be determined by mutual agreement. Submission to the Sponsor for review and comment is required prior to submission to the publisher. This requirement should not be construed as a mean of restricting publication, but is intended solely to ensure concurrence regarding data, evaluations, and conclusions, and to provide an opportunity to share with the Investigator any new or unpublished information of which he or she may be unaware.

APPENDIX

1. Classification of Adverse Events using the NIH Common Terminology Criteria for Adverse Events (CTCAE), Version 4.0.

REFERENCES

1. Chen C, Huang J, Cheng Z, et al. Favipiravir versus Arbidol for COVID-19: A Randomized Clinical Trial. *medRxiv.* 2020:2020.2003.2017.20037432.
2. Wu Z, McGoogan JM. Characteristics of and Important Lessons From the Coronavirus Disease 2019 (COVID-19) Outbreak in China: Summary of a Report of 72 314 Cases From the Chinese Center for Disease Control and Prevention. *JAMA.* 2020.

3. Delang L, Abdelnabi R, Neyts J. Favipiravir as a potential countermeasure against neglected and emerging RNA viruses. *Antiviral research.* 2018;153:85-94.
4. Furuta Y, Komeno T, Nakamura T. Favipiravir (T-705), a broad spectrum inhibitor of viral RNA polymerase. *Proceedings of the Japan Academy Series B, Physical and biological sciences.* 2017;93(7):449-463.
5. Cai Q YM, Liu D, Chen J, Shu D, Xia J, . Experimental Treatment with Favipiravir for COVID-19: An Open-Label Control Study. 2020.
6. Savarino A, Di Trani L, Donatelli I, Cauda R, Cassone A. New insights into the antiviral effects of chloroquine. *The Lancet Infectious diseases.* 2006;6(2):67-69.
7. Wang M, Cao R, Zhang L, et al. Remdesivir and chloroquine effectively inhibit the recently emerged novel coronavirus (2019-nCoV) in vitro. *Cell research.* 2020;30(3):269-271.
8. Marmor MF, Kellner U, Lai TY, Melles RB, Mieler WF. Recommendations on Screening for Chloroquine and Hydroxychloroquine Retinopathy (2016 Revision). *Ophthalmology.* 2016;123(6):1386-1394.
9. Gao J, Tian Z, Yang X. Breakthrough: Chloroquine phosphate has shown apparent efficacy in treatment of COVID-19 associated pneumonia in clinical studies. *Bioscience trends.* 2020;14(1):72-73.
10. McCreary EK, Pogue JM, Pharmacists obotSoID. COVID-19 Treatment: A Review of Early and Emerging Options. *Open Forum Infectious Diseases.* 2020.
11. Yao X, Ye F, Zhang M, et al. In Vitro Antiviral Activity and Projection of Optimized Dosing Design of Hydroxychloroquine for the Treatment of Severe Acute Respiratory Syndrome Coronavirus 2 (SARS-CoV-2). *Clinical infectious diseases : an official publication of the Infectious Diseases Society of America.* 2020.
12. Liu J, Cao R, Xu M, et al. Hydroxychloroquine, a less toxic derivative of chloroquine, is effective in inhibiting SARS-CoV-2 infection in vitro. *Cell discovery.* 2020;6:16.
13. Plaquenil-Hydroxychloroquine Sulfate package insert. . St. Michael, Barbados: Concordia Pharmaceuticals Inc;. 2015.
14. Avigan- Favipiravir package insert. Tokyo, Japan: FujiFilm Toyama Chemical Co. 2019.
15. Jallouli, M., Galicier, L., Zahr, N. and (2015), Determinants of Hydroxychloroquine Blood Concentration Variations in Systemic Lupus Erythematosus. Arthritis & Rheumatology, 67: 2176-2184. doi:10.1002/ art.39194

16. Borba MGS, Val FFA, Sampaio VS, et al. Effect of High vs Low Doses of Chloroquine Diphosphate as Adjunctive Therapy for Patients Hospitalized With Severe Acute Respiratory Syndrome Coronavirus 2 (SARS-CoV-2) Infection: A Randomized Clinical Trial. *JAMA Netw Open.* 2020;3(4.23):e208857.

17. Avigan- Favipiravir package insert. Tokyo, Japan: FujiFilm Toyama Chemical Co. 2019.

18. Ling Lin, Lianfeng Lu, Wei Cao & Taisheng Li (2020) Hypothesis for potential pathogenesis of SARS-CoV-2 infection–a review of immune changes in patients with viral pneumonia, Emerging Microbes & Infections, 9:1, 727-732, DOI: 10.1080/22221751.2020.1746199

19. Furuta Y, Komeno T, Nakamura T. Favipiravir (T-705), a broad spectrum inhibitor of viral RNA polymerase. Proceedings of the Japan Academy Series B, Physical and biological sciences. 2017;93(7):449-463.

Classification of Adverse Events using the NIH Common Terminology Criteria for Adverse Events (CTCAE), Version 4.0.

	**Grade**				
**Adverse Event**	**1**	**2**	**3**	**4**	**5**
Urticaria	Urticarial lesions covering <10% BSA; topical intervention indicated	Urticarial lesions covering 10 - 30% BSA; oral intervention indicated	Urticarial lesions covering >30% BSA; IV intervention indicated	-	-
Definition	A disorder characterized by an itchy skin eruption characterized by wheals with pale interiors and well-defined red margins.				
Bronchospasm	Mild symptoms; intervention not indicated	Symptomatic; medical intervention indicated; limiting instrumental ADL	Limiting self-care ADL; oxygen saturation decreased	Life-threatening respiratory or hemodynamic compromise; intubation or urgent intervention indicated	Death
Definition	A disorder characterized by an adverse local or general response from exposure to an allergen.				
Diarrhea	Increase of <4 stools per day	Increase of 4 - 6 stools per	Increase of >=7 stools per day over	Life-threatening consequences;	Death

	Grade				
**Adverse Event**	**1**	**2**	**3**	**4**	**5**
	over baseline; mild increase in ostomy output compared to baseline	day over baseline; moderate increase in ostomy output compared to baseline	baseline; incontinence; hospitalization indicated; severe increase in ostomy output compared to baseline; limiting self-care ADL	urgent intervention indicated	
Dyspnea	Shortness of breath with moderate exertion	Shortness of breath with minimal exertion; limiting instrumental ADL	Shortness of breath at rest; limiting self care ADL	Life-threatening consequences; urgent intervention indicated	Death
Definition	A disorder characterized by an uncomfortable sensation of difficulty breathing.				
Tongue Edema	Asymptomatic or mild symptoms; clinical or diagnostic observations only; intervention not indicated	Moderate; minimal, local or noninvasive intervention indicated; limiting age appropriate instrumental ADL	Severe or medically significant but not immediately life-threatening; hospitalization or prolongation of existing hospitalization indicated; disabling; limiting self-care ADL	Life-threatening consequences; urgent intervention indicated	Death
Definition					
Local skin necrosis at the injection site	Asymptomatic or mild symptoms; clinical or diagnostic observations only; intervention not indicated	Moderate; minimal, local or noninvasive intervention indicated; limiting age appropriate instrumental ADL	Severe or medically significant but not immediately life-threatening; hospitalization or prolongation of existing hospitalization indicated; disabling; limiting self-care ADL	Life-threatening consequences; urgent intervention indicated	Death
Definition					
Diarrhea	Increase of <4 stools per day over baseline; mild increase in ostomy output compared to baseline	Increase of 4 - 6 stools per day over baseline; moderate increase in ostomy output compared to baseline; limiting instrumental ADL	Increase of >=7 stools per day over baseline; hospitalization indicated; severe increase in ostomy output compared to baseline; limiting self care ADL	Life-threatening consequences; urgent intervention indicated	Death

	Grade				
**Adverse Event**	**1**	**2**	**3**	**4**	**5**
Definition	A disorder characterized by an increase in frequency and/or loose or watery bowel movements.				
Dysgeusia	Altered taste but no change in diet	Altered taste with change in diet (e.g., oral supplements); noxious or unpleasant taste; loss of taste	-	-	-
Definition:	A disorder characterized by abnormal sensual experience with the taste of foodstuffs; it can be related to a decrease in the sense of smell.				
Nausea	Loss of appetite without alteration in eating habits	Oral intake decreased without significant weight loss, dehydration or malnutrition	Inadequate oral caloric or fluid intake; tube feeding, TPN, or hospitalization indicated	-	-
Definition	A disorder characterized by a queasy sensation and/or the urge to vomit.				
Vomiting	Intervention not indicated	Outpatient IV hydration; medical intervention indicated	Tube feeding, TPN, or hospitalization indicated	Life-threatening consequences	Death
Definition:	A disorder characterized by the reflexive act of ejecting the contents of the stomach through the mouth.				
Abdominal Pain	Mild pain	Moderate pain; limiting instrumental ADL	Severe pain; limiting self care ADL	-	-
Definition:	A disorder characterized by a sensation of marked discomfort in the abdominal region.				
Headache	Mild pain	Moderate pain; limiting instrumental ADL	Severe pain; limiting self-care ADL	-	-
Definition:	A disorder characterized by a sensation of marked discomfort in various parts of the head, not confined to the area of distribution of any nerve.				
Depression	Mild depressive symptoms	Moderate depressive symptoms; limiting instrumental ADL	Severe depressive symptoms; limiting self-care ADL; hospitalization not indicated	Life-threatening consequences, threats of harm to self or others; hospitalization indicated	Death

	Grade				
**Adverse Event**	**1**	**2**	**3**	**4**	**5**
Insomnia	Mild difficulty falling asleep, staying asleep or waking up early	Moderate difficulty falling asleep, staying asleep or waking up early	Severe difficulty in falling asleep, staying asleep or waking up early	-	-
Definition	A disorder characterized by difficulty in falling asleep and/or remaining asleep.				
Psychosis	Mild psychotic symptoms	Moderate psychotic symptoms (e.g., disorganized speech; impaired reality testing)	Severe psychotic symptoms (e.g., paranoid; extreme disorganization); hospitalization not indicated	Life-threatening consequences, threats of harm to self or others; hospitalization indicated	Death
Definition	A disorder characterized by personality change, impaired functioning, and loss of touch with reality. It may be a manifestation of schizophrenia, bipolar disorder or brain				
Depression	Mild depressive symptoms	Moderate depressive symptoms; limiting instrumental ADL	Severe depressive symptoms; limiting self care ADL; hospitalization not indicated	Life-threatening consequences, threats of harm to self or others; hospitalization indicated	Death
Definition	A disorder characterized by melancholic feelings of grief or unhappiness.				
Mania	Mild manic symptoms (e.g., elevated mood, rapid thoughts, rapid speech, decreased need for sleep)	Moderate manic symptoms (e.g., relationship and work difficulties; poor hygiene)	Severe manic symptoms (e.g., hypomania; major sexual or financial indiscretions); hospitalization not indicated	Life-threatening consequences, threats of harm to self or others; hospitalization indicated	Death
Definition	A disorder characterized by excitement of psychotic proportions manifested by mental and physical hyperactivity, disorganization of behavior and elevation of mood.				
Electrocardiogram QT corrected interval prolonged	Average QTc 450 - 480 ms	Average QTc 481 - 500 ms	Average QTc >= 501 ms; >60 ms change from baseline	Torsade de pointes; polymorphic ventricular tachycardia; signs/symptoms of serious arrhythmia	
Definition	A finding of a cardiac dysrhythmia characterized by an abnormally long corrected QT interval.				
Skin and	Asymptomatic or	Moderate;	Severe or medically	Life-threatening	Death

	Grade				
**Adverse Event**	**1**	**2**	**3**	**4**	**5**
subcutaneous tissue disorders - Other, specify	mild symptoms; clinical or diagnostic observations only; intervention not indicated	minimal, local or noninvasive intervention indicated; limiting age appropriate instrumental ADL	significant but not immediately life-threatening; hospitalization or prolongation of existing hospitalization indicated; disabling; limiting self-care ADL	consequences; urgent intervention indicated	
